# Methylglyoxal Levels in Human Colorectal Precancer and Cancer: Analysis of Tumor and Peritumor Tissue

**DOI:** 10.3390/life11121319

**Published:** 2021-11-30

**Authors:** Chu-Kuang Chou, Po-Chun Yang, Pei-Yun Tsai, Hsin-Yi Yang, Kun-Feng Tsai, Tsung-Hsien Chen, Kai-Sheng Liao, Chi-Yi Chen, Jen-Ai Lee

**Affiliations:** 1 Division of Gastroenterology and Hepatology, Ditmanson Medical Foundation Chia-Yi Christian Hospital, Chiayi 60002, Taiwan; a250624545@hotmail.com (P.-C.Y.); 5137ccy@gmail.com (C.-Y.C.); 2 Clinical Trial Center, Ditmanson Medical Foundation Chia-Yi Christian Hospital, Chiayi 60002, Taiwan; 3 School of Pharmacy, College of Pharmacy, Taipei Medical University, Taipei 11031, Taiwan; kellyrabbit324@gmail.com (P.-Y.T.); jenai@tmu.edu.tw (J.-A.L.); 4 Clinical Medicine Research Center, Ditmanson Medical Foundation Chia-Yi Christian Hospital, Chiayi 60002, Taiwan; cych13018@gmail.com; 5 Gastroenterology and Hepatology Section, Department of Internal Medicine, An-Nan Hospital, China Medical University, Tainan 70965, Taiwan; tsai.kf@gmail.com; 6 Department of Medical Sciences Industry, Chang Jung Christian University, Tainan 71101, Taiwan; 7 Department of Internal Medicine, Ditmanson Medical Foundation Chia-Yi Christian Hospital, Chiayi 60002, Taiwan; cych13794@gmail.com; 8 Department of Pathology, Ditmanson Medical Foundation Chia-Yi Christian Hospital, Chiayi 60002, Taiwan; 07455@cych.org.tw; 9 Department of Nursing, Chung-Jen Junior College of Nursing, Health Sciences and Management, Da-Lin, Chiayi 62241, Taiwan

**Keywords:** human colorectal cancer, methylglyoxal, precancerous and cancerous, tumor and peritumor tissue

## Abstract

Colorectal cancer (CRC) is one of the most common cancers worldwide and its incidence is increasing; therefore, an understanding of its oncogenic mechanisms is critical for improving its treatment and management. Methylglyoxal (MGO) has a highly reactive aldehyde group and has been suggested to play a role in oncogenesis. However, no standardized data are currently available on MGO levels in colorectal precancerous and cancerous lesions. We collected 40 matched colorectal tumor and peritumor tissues from patients with low-grade dysplasia (LGD), high-grade dysplasia (HGD), and invasive cancer (IC). MGO levels increased between LGD, HGD, and IC tumor tissues (215.25 ± 39.69, 267.45 ± 100.61, and 587.36 ± 123.19 μg/g protein, respectively; *p* = 0.014). The MGO levels in peritumor tissue increased and were significantly higher than MGO levels in tumor tissue (197.99 ± 49.40, 738.09 ± 247.87, 933.41 ± 164.83 μg/g protein, respectively; *p* = 0.002). Tumor tissue MGO levels did not correlate with age, sex, underlying disease, or smoking status. These results suggest that MGO levels fluctuate in progression of CRC and warrants further research into its underlying mechanisms and function in tumor biology.

## 1. Introduction

Colorectal cancer (CRC) is one of the most important public health concerns and results in the most cancer-related deaths worldwide [1]. A majority of the adenomas of CRCs develop from precancerous adenoma with low-grade dysplasia (LGD), which typically progress to high-grade dysplasia (HGD) and invasive cancer (IC) [2]. Inflammatory bowel disease is an important risk factor that is involved in the development of CRC, which is a multifactorial disease resulting from a combination of genetic, environmental, and lifestyle risk factors [3,4].

The formation and accumulation of methylglyoxal (MGO) is associated with age-related diseases such as atherosclerosis, cancer, metabolic syndrome and neurodegenerative disorders [5]. Fat oxidation and glyceraldehyde 3-phosphate pathways and glycolysis pathways may be important sources of MGO production [6]. MGO is the most potent glycating agent in humans and reacts with proteins to generate advanced glycation end products (AGEs) [7], resulting in protein dysfunction, AGE receptor activation, chronic inflammation, oxidative stress, and oncogenesis [8]. The accumulation of AGEs is due to cellular processes that cause mitochondrial dysfunction, genomic instability, loss of proteostasis, inflammation, and cellular senescence [9]. In addition, MGO reacts with DNA to form glycated DNA, which generates excessive reactive oxygen species (ROS) [10], leading to cell toxicity, apoptosis or necrosis [11]. MGO abduction is higher in human CRC tissue than in normal tissue [12,13] and may inhibit cancer growth via eliciting direct toxicity, promoting oncogenesis by enhancing AGEs, or other unknown pathways [14,15]. Cancer cells alter their metabolism to enhance proliferation, growth and survival by increasing their energy production from glycolysis [16], known as the Warburg effect, which results in MGO accumulation. 

However, molecular analyses of the association between MGO and CRC have yielded contradictory results [17,18,19,20]. CRC cells with different MGO concentrations reduced cell viability in a time- and dose-dependent manner, induced apoptosis, and inhibited cell migration and invasion [19]. The g cancer cell growth was reduced in the peritoneum of MGO-treated murine CRC model [19,20]. Glyoxalase I (GLOI), which catalyzes MGO metabolism, is associated with the progression of human malignancies and MGO exhibited a greater inhibitory effect when combined with short hairpin RNAs (shRNAs), silencing GLOI [20]. Furthermore, shRNA-mediated GLOI depletion without MGO administration inhibited CRC proliferation and migration, induced cell line apoptosis, and inhibited tumor growth in a murine model [21]. Conversely, Chiavarina et al., [12] found that an increased accumulation of MGO adducts can cause an inflammatory response, which is significantly related to tumor-promotion in patients with advanced CRC. In addition, intraperitoneal MGO administration in BALB/c mice with CT26 isografts enhanced CRC growth [18]. MGO-induced carbonyl stress can also enhance the degree of malignancy of tumor cells and increased the expression of tumor proliferation and migration markers CD29, CD44, and Msi-1 [18].

To date, the variation in the MGO levels throughout oncogenesis in human CRC tissue has not been evaluated. Previous resection techniques for large, early colorectal neoplasms (LGD and HGD) resulted in small, fragmented pieces, making it difficult to distinguish between normal and cancerous tissue and quantify their respective MGO levels. However, the development of endoscopic submucosal dissection techniques allows for large early colorectal neoplasms to be resected with adequate peritumor tissue [22], which permits the analysis of MGO levels in both tumor and peritumor tissue. In this study, we quantified MGO levels from precancerous to cancerous colorectal lesions, providing insight into its potential role in colorectal carcinogenesis, and possible pathogenic mechanisms.

## 2. Materials and Methods

### 2.1. Patient Selection and Data Collection

Patients with colorectal neoplasms, including pre-cancerous and cancerous lesions, were enrolled between June 2014 and June 2016. A total of 40 primary tumors were collected from patients diagnosed with CRC who underwent surgery at Chiayi Christian Hospital (Chiayi, Taiwan). The study was approved by the Institutional Review Board of Chiayi Christian Hospital (protocol code 102057). Demographic data, including age, gender, diabetes mellitus, hypertension, and smoking history, were collected by reviewing patient charts and from questionnaires. CRC staging was carried out according to the 7th edition American Joint Committee on Cancer (AJCC) criteria [23] and determined according to pathological diagnosis for patients whose cancerous lesions were fully resected. 

### 2.2. Tissue Collection

Colorectal tumor and peritumor mucosa tissue were collected from forty participants during scheduled procedures conducted according to the current guidelines for the management of colorectal neoplasms [24].

**Figure 1:**
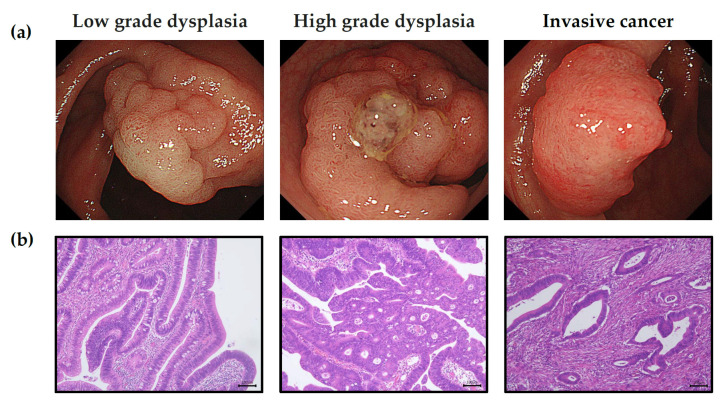
Classification of colorectal tumor mucosa tissues by (**a**) white-light imaging endoscopy, and (**b**) Hematoxylin and eosin (H&E) staining of tumor mucosa tissue samples. Tumor tissues are classified as low-grade dysplasia, high-grade dysplasia, and invasive cancer (T1 colorectal cancer), sequentially. Scale bar = 100 microns.

**Figure 2:**
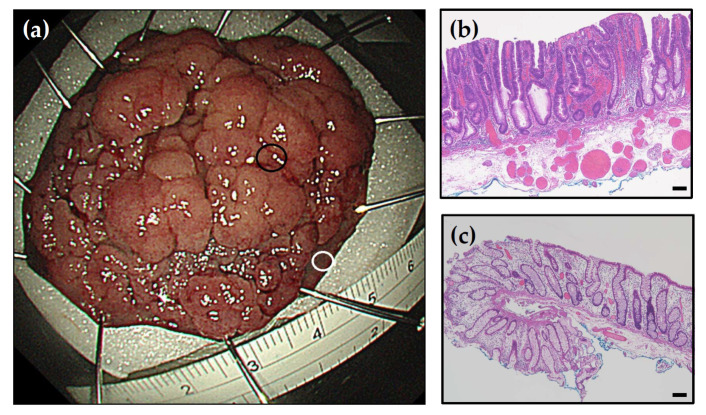
Endoscopic submucosal dissection. (**a**) Tumor tissue was dissected from the specimen (black circle) and peritumor tissue was identified and collected (white circle). (**b**) Hematoxylin and eosin (H&E) staining of tumor sections. (**c**) H&E staining of peritumor tissue sections. Scale bar = 100 microns.

Endoscopic submucosal dissection was used for the resection of LGD, HGD, and T1 IC (invasive cancer) tumor tissues, since there was an adequate peritumor safety margin [22,25] (Figure 1a). The colon wall consists of an inner mucosal layer (location of early colorectal neoplasms), an outer muscle layer, and a submucosal layer. After defining the resection area with an adequate safety margin, the submucosal layer was dissected to separate the mucosa from the muscle layer, enabling the resection of a large area of the mucosa and submucosa consisting of both the tumor and peritumor safety margins. The lateral peritumor safety margin was approximately 0.5–1 cm (Figure 2). If the IC was not suitable for endoscopic submucosal dissection, we used biopsy forceps to directly resect the tumor and peritumor tissue.

Once the lesion had been resected, small sections of the tumor and peritumor tissue were dissected; washed with normal saline to remove blood, stool, and other contaminants; placed into Eppendorf tubes on ice; and stored at −80 °C until further analysis. Most of the main lesions were stored in 4% formaldehyde solution and sent to the pathology department for further histopathological diagnosis.

### 2.3. Pathology

Hematoxylin and eosin (H&E) staining was used on all dissected tissue samples to determine standard pathologic staging (i.e., tumor type, differentiation, budding, grade, invasion depth, lymphovascular invasion, and perineural invasion). Adenoma with LGD, HGD, or IC was diagnosed according to the World Health Organization’s classifications [26] (Figure 1b). Adenoma was defined as dysplastic endothelium, low- or high-grade dysplasia were defined by both cytological and architectural features, and invasive cancers were defined by the presence of neoplastic glands penetrating from the muscularis mucosa into the submucosa.

We followed Vienna classification [27]. Dysplasia was defined as neoplastic epithelial proliferation with the potential to become invasive. A distinction between LGD and HGD was conventionally based on features as whether the neoplastic nuclei were limited primarily to the lower or upper halves of the cells in the glands. The LGD looks close to normal tissue with some epithelial proliferation. The picture of LGD showed stratification of nuclei that do not reach the luminal cell surface, mildly elongated and dysplastic nuclei and mitotic activity with mild loss of polarity.

### 2.4. Total Protein Assay

Human colonic tissue was homogenized with phosphate buffered saline (PBS) (Sigma-Aldrich, St. Louis, MO, USA), diluted four-fold, and subjected to analysis with a commercial protein assay kit (Bio-Rad, Hercules, CA, USA) according to the manufacturer’s instructions. A working reagent was prepared with Bicinchoninic Acid (BCA) reagents A and B at a ratio of 50:1 (*v/v*). A stock bovine serum albumin (BSA) solution (2 mg/mL) was diluted to 25, 125, 250, 750, 1,250, 1,750, and 2,000 μg/mL to produce a standard curve. Both the sample and standards solution (25 µL) were placed into Eppendorf tubes with 200µL working buffer, shaken for 30 sec, and incubated at 37 °C for 30 min and at room temperature for 3 min. The sample and standard solution (200 µL) was then transferred onto a 96 well plate and its optical density was measured at 562 nm.

### 2.5. Quantification of MGO Concentrations by Fluorescent HPLC Analysis

Tissue MGO levels were determined using a fluorescent high performance liquid chromatography (HPLC) analysis, as described previously [28]. All HPLC equipment was purchased from Hitachi (Tokyo, Japan). Colon homogenates were added to 2 μL of ammonium chloride buffer (pH 10; Sigma-Aldrich, St. Louis, MO, USA) to create an alkaline environment for derivatization. The derivative reagent (50 µL of 7.5 × 10^-4^ M 5,6-diamino-2,4-dihydroxy-pyrimidine; Sigma-Aldrich, St. Louis, MO, USA) was added, the mixture was incubated at 60 °C for 30 min in the dark, and the reaction was stopped by adding 500 µL of 0.01 M citric acid buffer (pH 6; Sigma-Aldrich, St. Louis, MO, USA). The mixture was vortexed, spun down, incubated on ice for 10 min, and centrifuged at 12,000 rpm for 10 min at 4 °C. The supernatant was filtered (0.45 µm) to remove impurities, layered to a ODS column (250 mm × 4.6 mm i.d.; 5-μm particle size; Biosil Chemical, Taipei, Taiwan), separated at 33 °C, with acetonitrile (Merck KGaA, Darmstadt, Germany) and 0.01 M citric acid buffer (3:97, *v/v*; Sigma-Aldrich, St. Louis, MO, USA) as the mobile phase, at a flow rate of 0.7 mL/min, and excitation/emission wavelengths of 330/500 nm. All samples were analyzed within 24 h.

Various concentrations (25, 50, 100, 200, 400, 600, 800, and 1000 µg/L) of MGO (20 µL of 40% aqueous solution; Sigma-Aldrich, St. Louis, MO, USA) were used to produce a calibration curve plotted according to the peak area ratio of MGO. Tissue samples were added to the different concentrations of the 20 µL MGO standard (0, 300, and 600 µg/L) and analyzed by fluorescent HPLC described above. Precision was determined using relative standard deviation (RSD) and accuracy was expressed in terms of calculated recovery.

### 2.6. Immunohistochemistry

Resected MGO colorectal neoplasm were sliced into 3-μm sections, embedded in paraffin, and analyzed by immunohistochemical staining to determine MGO localization. LGD, HGD, or IC tumor tissue sections were deparaffinized with xylene (Sigma, St. Louis, MO, USA), rehydrated in a series of alcohol (Sigma, St. Louis, MO, USA), and rinsed with diH_2_O and 1× Tris-buffered saline (TBS), following standard protocol. Antigen retrieval was performed in antigen unmasking solutions (Vector, Burlingame, CA, USA), samples were placed in a pressure cooker (solution temperature ≈ 120°) for 1min, and run under cold water for 14 min.

After blocking the samples with 3% H_2_O_2_ (Sigma, St. Louis, MO, USA) for 10 min, the slides were washed with PBS. Thereafter, the slides were incubated with Blocking Reagent A (Nichirei Biosciences, Tokyo, Japan) for 60 min at room temperature. Anti-MGO monoclonal antibody (STA-001, Cell biolabs, San Diego, CA, USA) was diluted 50-fold and incubated with the samples overnight. The samples were then washed with PBS and incubated with Blocking Reagent B (Nichirei Biosciences, Tokyo, Japan) for 10 min at room temperature. The samples were then incubated with a secondary antibody (reduced Fab antibody fragments of goat anti-mouse IgG conjugated with a labeled polymer prepared by combining amino acid polymer with multiple molecules of peroxidase) for 10 min at room temperature. Thereafter the samples were dried, co-cultured with chromogen/substrate reagent (Thermo, Waltham, MA, USA), and incubated in the dark at room temperature for 10 min. Counterstaining with hematoxylin was performed and a quantitative image analysis of the immunohistochemical stains was performed by pathologists, and the proportion of cytoplasmic and nuclear MGO in tumor cells was determined.

### 2.7. Statistical Analyses

All statistical analyses were performed using SPSS for Windows version 21.0 (IBM Corp., Armonk, NY, USA). Continuous variables were expressed as means ± standard error, and categorical data were expressed as percentages. Comparisons of continuous data were made between groups using Mann-Whitney U tests, and categorical data were compared using the chi-square test or Fisher’s exact test. Multiple comparisons were made using the Kruskal-Wallis test with Dunn’s post-hoc test. Correlations between parameters were calculated using Pearson’s correlation coefficient and univariate regression analysis with the least-squares method. Statistical significance was set at 95 % (*p* < 0.05).

## 3. Results

### 3.1. Demographic Data for the Different Groups of Colorectal Neoplasms

In total, forty patients were enrolled in this study and divided into three groups according to their pathology: adenoma with LGD (n = 11), adenoma with HGD (n = 14), and IC (n = 15). Six out of the fifteen patients with invasive cancer were classified as stage I, four as stage II, and five as stage III. There were no significant differences in age, sex, diabetes, hypertension, or smoking status between the groups (Table 1). 

### 3.2. Fluorescent HPLC Results and Method Validation in Human Colon Samples

**Figure 3:**
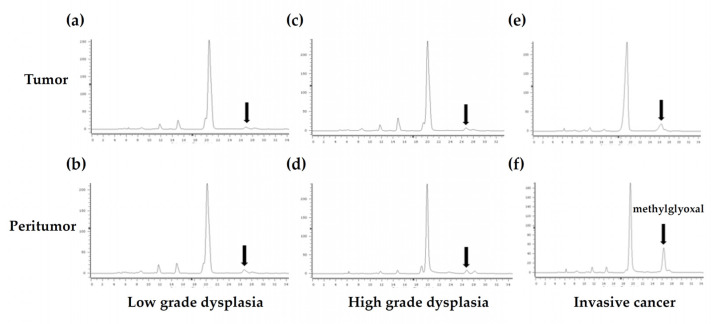
Detection of methylglyoxal in various stages of colorectal cancer using fluorescent high performance liquid chromatography: (**a**) low- grade dysplasia tumor tissue, (**b**) low-grade dysplasia peritumor tissue, (**c**) high-grade dysplasia tumor tissue, (**d**) high-grade dysplasia peritumor tissue, (**e**) invasive cancer tissue, (**f**) invasive cancer peritumor tissue. Arrow indicates the methylglyoxal peak. The reaction between methylglyoxal and the derivatization agent (DDP) in the human colorectal tissue produces a fluorescent product, which allows low concentrations of methylglyoxal to be detected.

The MGO peak appeared at 26 min and the standard curve indicated a good linear relationship (R^2^ = 0.9997). All chromatograms for colorectal LGD, HGD, and IC samples are shown in Figure 3. The precision (RSD) and accuracy (recovery) of MGO determination in human colon samples are shown in Table S1. Intra-assay values indicated an accuracy of 115.14–119.93% and a precision of 2.70–10.84% (RSD) for triplicate measurements, whilst inter-assay values indicated an accuracy of 106.49–113.2 % and a precision of 2.57–7.98% (RSD) for triplicate measurements.

### 3.3. MGO Levels in Tumor and Peritumor Human Colon Tissue

**Figure 4:**
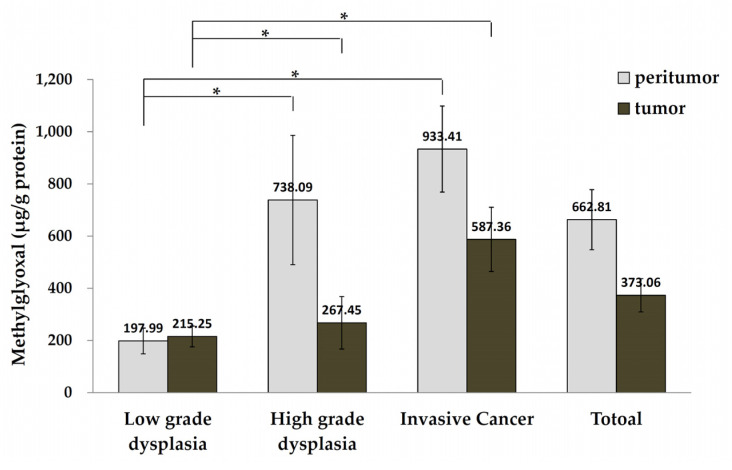
Comparison of methylglyoxal levels in tumor and peritumor tissue from patients with low-grade dysplasia, high-grade dysplasia, and invasive cancer (* *p* < 0.05). Nonparametric statistics (Kruskal-Wallis test), post-hoc test: Dunn test.

MGO levels increased between LGD, HGD, and IC tumor tissues (215.25 ± 39.69, 267.45 ± 100.61, and 587.36 ± 123.19 μg/g protein, respectively; *p* = 0.014). Interestingly, MGO levels between LGD, HGD and IC peritumor tissues increased and were significantly higher than MGO levels in tumor tissues (197.99 ± 49.40, 738.09 ± 247.87, 933.41 ± 164.83 μg/g protein, respectively; *p* = 0.002; Figure 4). MGO levels in tumor tissue from the LGD group were slightly lower than that of the peritumor tissue in the LGD group (215.25 ± 39.69 μg/g vs 197.99 ± 49.40 μg/g of protein, respectively; *p* = 0.491). MGO levels in HGD and IC tumor tissues were substantially lower than in peritumor tissues (738.09 ± 247.88 vs 267.45 ± 100.61 μg/g of protein, respectively; *p* = 0.089; and 933.41 ± 164.83 vs 587.36 ± 123.19 μg/g of protein, respectively; *p* = 0.085) (Figure 4). 

### 3.4. Evidence of MGO Dicarbonyl Stress in Human CRC Tumors

**Figure 5:**
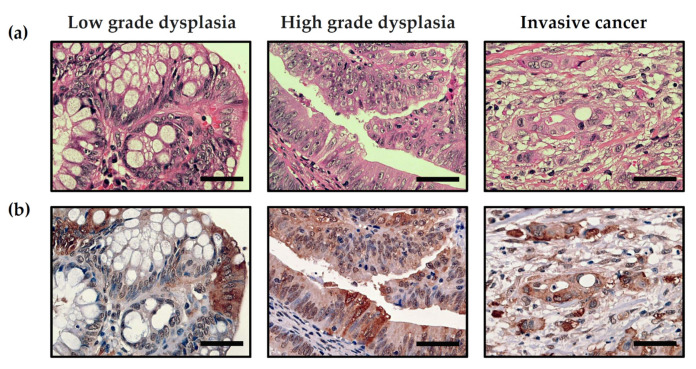
Evaluation of methylglyoxal (MGO) levels in low-grade dysplasia, high-grade dysplasia, and invasive cancer (T1) colorectal cancer tissues: (**a**) Hematoxylin and eosin (H&E) staining, (**b**) Immunohistochemical staining. Scale bar = 100 microns.

IHC staining results indicated that MGO levels in LGD, HGD and IC tumor tissues slightly increased gradually (Figure 5). The proportion of cytoplasm stained by MGO in LGD, HGD, and IC tumor tissues were 0.01 ± 0.02, 0.05 ± 0.07, and 0.07 ± 0.12, respectively (*p* = 0.138). Additionally, the proportion of nuclei stained by MGO in LGD, HGD, and IC tumor tissues were 0.20 ± 0.31, 0.34 ± 0.37 and 0.45 ± 0.33, respectively (*p* = 0.237). There was a positive correlation between the proportion of cytoplasmic staining and MGO levels (0.509, *p* < 0.01).

### 3.5. Association of Tissue MGO Levels with Age and Underlying Diseases

**Figure 6:**
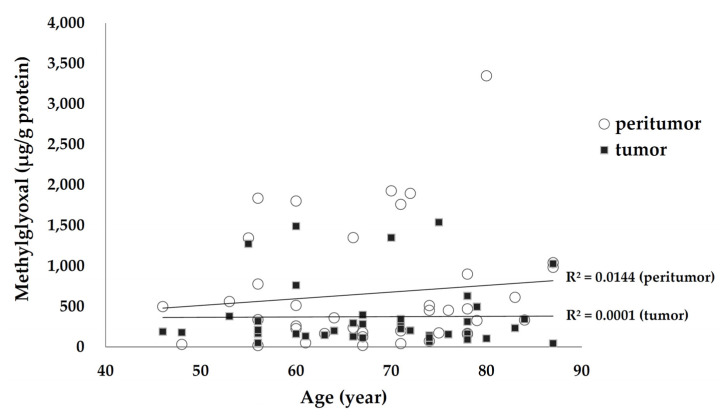
Comparison between age and methylglyoxal levels in tumor and peritumor tissue.

Tissue MGO levels were not related to age in either the peritumor tissue (β-value of linear regression = 0.12, *p* = 0.461) or tumor tissue (β-value = 0.01, *p* = 0.945; Figure 6). Moreover, MGO levels in both groups did not differ according to diabetes, hypertension co-morbidity, smoking status, or sex. 

## 4. Discussion

Our results provide novel insights into the function of MGO in colorectal oncogenesis, which could help identify potential treatment targets or preventive strategies for patients with early-stage CRC. MGO levels increased between LGD, HGD, and IC tumor tissues, and MGO levels in peritumor tissues were significantly higher than in tumor tissues. MGO levels in tumor tissues did not correlate with age, sex, underlying disease, or smoking status. 

Patients with LGD have a lower risk of developing cancer than patients with HGD. LGD resolves in 38–75% of the cases and persists in 19–50% of cases [29]. Among the patients with LGD, 0–23% show malignant changes within an average of 10–48 months from initial diagnosis [30,31,32,33,34]. Additionally, patients with LGD have a lower risk of malignant transformation (3–9%) [32,33]. In a recent Dutch national study, it was found that recurrent LGD at a follow-up colonoscopy is a risk factor for developing advanced neoplasia in patients with inflammatory bowel disease (the incidence rate of recurrent LGD vs non-recurrent LGD: 22.7 vs 14.0 per 1,000 patient-years) [35]. Previous resection techniques for large early colorectal neoplasms (LGD and HGD) resulted in small, fragmented pieces, making it difficult to distinguish between peritumor and tumor tissue and quantify their respective MGO levels. However, the development of endoscopic submucosal dissection techniques allows large early colorectal neoplasms to be resected with adequate peritumor tissue [22], permitting the analysis of MGO levels in both tumor and peritumor tissue. Here, we quantified MGO levels in colorectal precancerous to cancerous lesions, providing insight into its potential role in colorectal carcinogenesis and a new tool to understand its underlying mechanisms.

Many cytokines that are produced by intestinal immune cells during or in response to local inflammation in the colon and rectum, can stimulate complex interactions between different cell types in the intestinal environment, leading to acute inflammation [36]. Inflammation has been shown induce the occurrence and progression of CRC [37]. MGO accumulation can promote inflammation and contribute to endothelial dysfunction. The dual-role of MGO in tumor progression and its anti-cancer effect was initially attributed to MGO cytotoxicity, and its subsequent pro-tumorigenic effect was observed in several types of cancer, including CRC [38]. Normal cells use mitochondrial oxidative respiration with oxygen availability to produce energy. However, cancerous cells have a high rate of glucose uptake, lactate secretion and oxygen availability. One of the main differences in cancer cell metabolism is represented by their preference to use anaerobic glycolysis to produce adenosine triphosphate (ATP), but this process produces less ATP than glucose fermentation [39]. Cancer cells prefer glucose fermentation to lactate because this process is 10–100 times faster than complete mitochondrial glucose oxidation [40]. Compared with glucose, glucose-derived glycolysis intermediates, especially MGO, produce much more glycated proteins in a faster way [15]. This leads to AGE accumulation and receptor for AGE (RAGE) pathway activation which contributes to cancer pathogenesis by fostering tissue and cellular dysfunction [41]. This is the first study to compare human colorectal cancerous and precancerous tissue MGO levels, and our results indicated MGO levels is higher in peritumor tissue than in tumor tissue. Moreover, MGO levels clearly increased in a sequence corresponding to oncogenesis, during the progression of LGD to HGD to IC, suggesting that MGO plays a role in cancer progression. HPLC results can clearly distinguish the difference in MGO accumulation levels between LGD, HGD and IC segments. In the IHC stains, we observed a trend in MGO cytoplasmic staining in LGD, HGD and IC segments. However, due to the limited number of cases, there was no statistically significant difference in the proportion of MGO-stains between LGD, HGD and IC sections in the cytoplasmic or nuclear tumor tissue stains. 

MGO has significant selectivity for malignant proliferating cells. The proliferation/apoptosis is progressive from LGD, HGD to invasive cancer. Previous study of colon cancer has shown possible effect of MGO on apoptosis and proliferation through cell line study [12,18,19]. MGO induces the inhibition of cell growth and toxicity of human leukemia 60 cells [42], inducing the accumulation of nucleic acid and protein adducts which leads to cell apoptosis [43]. Conversely, no significant inhibition of cell growth was found in mature neutrophils and lymphocytes (peripheral leucocytes) [42,43]. In addition, MGO treatment inhibited mitochondrial respiration of malignant cells, but had no inhibitory effect on the respiration of normal cells [32]. The anti-tumor effect of MGO is attributed to the inactivation of glyceraldehyde 3-phosphate dehydrogenase (GAPDH), which plays an important role in the high glycolytic capacity of malignant cells [44]. However, tumor growth was inhibited by single or continuous MGO administration in rodents inoculated with tumor cells [19,45]. 

In contrast, MGO protects gastrointestinal cancer cells from apoptosis by increasing the anti-apoptotic activity of endogenous MGO-modified heat shock protein (HSP) 27, through the inhibition of caspase-3 and 9 activation [46]. MGO-induced post-translational glycation of Hsp90 affects its activity with a consequent decrease in large tumor suppressor 1 (LATS1) expression, thereby promoting cancer cell growth and proliferation [47]. The accumulation of MGO adducts (e.g., argypirimidine) is found to be positively correlated with primary tumor staging, and the degree of dicarbonyl-induced stress is associated with CRC tumor aggressiveness [12]. MGO administration produces low-grade carbonyl stress that can lead to inflammation and oxidative stress, which is responsible for the deterioration of chemically induced colonic preneoplastic lesions in mice with colon cancer. Additionally, MGO induces colon cancer isograft growth by enhancing the expression/activation of proteins related to cell survival, proliferation, migration, and invasion [18]. 

MGO is a key contributor to diabetes-related complications and diabetes mellitus is a known risk factor for CRC [48]. Abnormal serum LDL/HDL ratio and fecal bile acid levels was reported exposure to MGO increased in azoxymethane-induced mice, which may play important roles in promoting CRC [18]. Metformin, typically used as a clinical anti-diabetic agent, can lower systemic MGO levels in a dose-dependent manner and h reduces the risk of CRC [49,50]. Plasma MGO levels are correlated with some systemic diseases, including diabetes [51], hypertension [52], renal failure [53] and septic shock [54]. We found that tissue MGO levels did not correlate with diabetes, smoking, or other underlying diseases; however, the case numbers are limited, and further investigation is necessary. Age is a risk factor for CRC and the incidence of CRC is higher among older adults [55]. However, our results indicated that MGO levels in colorectal tumor and peritumor tissue were not associated with age but rather to cancer grade or stage. In addition, we found that tissue MGO levels might be a reliable marker for cancer oncogenesis. These results suggest that MGO may promote CRC; however, no reliable epidemiological data has associated systemic or tissue MGO levels with CRC or compared MGO levels in CRC and precancerous lesions. 

The main limitation of our study is that, although we observed higher MGO levels in peritumor tissue than in tumor tissue, we did not harvest tissue located further from the tumor tissue, and therefore could not determine whether MGO levels would be different in normal tissue distant to the tumor. Hence, a more comprehensive analysis with tissue harvested from different areas of the colon is needed. Moreover, we did not examine the prognostic effect of MGO levels in tumor or peritumor tissue since our cohort only consisted of patients with precancerous and cancerous lesions. The survival of the patients with precancerous lesions was high and the local recurrence was low after lesion resection via colonoscopy. In this study, precancerous lesion growth in other sections of the colon was also very rare; thus, a larger sample size is required to establish any prognostic effect.

## 5. Conclusions

This is the first study to demonstrate that MGO levels gradually increased along with the different stages of CRC tumor tissue progression (LGD, HGD, and IC). Moreover, we found that MGO levels were higher in peritumor tissues than in tumor tissues. 

## Figures and Tables

**Table 1:** Study population demographics.

	Low-Grade Dysplasia	High-Grade Dysplasia	Invasive Cancer	Total	*p*-Value *
Number	11	14	15	40	
Age (years)	67.91 ± 3.17	70.50 ± 2.81	66.20 ± 2.83	68.18 ± 1.67	0.408
Sex					0.830
Female	4 (36.36)	5 (35.71)	4 (26.67)	13 (32.50)	
Male	7 (63.64)	9 (64.29)	11 (73.33)	27 (67.50)	
Diabetes mellitus	1 (9.09)	3 (21.43)	1 (6.67)	5 (12.50)	0.448
Hypertension	2 (18.18)	3 (21.43)	3 (20.00)	8 (20.00)	0.980
Smoking	1 (9.09)	2 (14.29)	5 (33.33)	8 (20.00)	0.250

* Non-parametric data (Kruskal-Wallis test), post-hoc test: Dunn test, chi-square test. Continuous data: mean ± SEM; categorical data: number (proportion).

## Data Availability

The data presented in this study are available on request from the corresponding author.

## Supplementary Materials

The following are available online at https://www.mdpi.com/article/10.3390/life11121319/s1, Table S1: Precision and accuracy of methylglyoxal determination method in human colon samples (n = 3).
