# Supplementary material for: Methylglyoxal Levels in Human Colorectal Precancer and Cancer: Analysis of Tumor and Peritumor Tissue
Source: Life (Basel). 2021 Nov 30;11(12):1319. doi: 10.3390/life11121319 (PMC8708054; doi:10.3390/life11121319)
Supplement: Supplementary file 1 [file life-11-01319-s001.zip › life-1438630-supplementary.pdf]

Supplementary Material

Methylglyoxal Levels in Human Colorectal Precancer and Cancer: Analysis of Tumor and Peritumor Tissue

Chu-Kuang Chou, Po-Chun Yang, Pei-Yun Tsai, Hsin-Yi Yang, Kun-Feng Tsai, Tsung-Hsien Chen, Kai-Sheng Liao, Chi-Yi Chen and Jen-Ai Lee

Table S1. Precision and accuracy of methylglyoxal determination method in human colon samples (n = 3).

		**Methylglyoxal Added (μg/L)**		
		0	300	600
Intra-assay	Measured (μg/L)	391.96 ± 42.47	572.33 ± 21.89	719.81 ± 19.43
	RSD (%)	10.84	3.82	2.70
	Recovery (%)	-	119.93	115.14
Inter-assay	Measured (μg/L)	385.89 ± 30.80	565.78 ± 16.32	731.30 ± 18.80
	RSD (%)	7.98	2.89	2.57
	Recovery (%)	-	113.22	106.49

RSD, Relative Standard Deviation
